# When the culprit lies outside the coronary artery: dual case report of coronary sinus of valsalva dissection presenting as STEMI

**DOI:** 10.3389/fcvm.2025.1670164

**Published:** 2025-09-22

**Authors:** Kun Chen, Mengjiao Yu, Youpeng Ling

**Affiliations:** Changde Hospital, Xiangya School of Medicine, Central South University, The First People’s Hospital of Changde City, Changde, China

**Keywords:** sinus of valsalva dissection, computed tomography angiography, ST-elevation myocardial infarction, coronary artery bypass grafting, intravascular ultrasound

## Abstract

Located sinus of Valsalva (SOV) dissection is a rare but critical condition that presents as inferior ST-segment elevation myocardial infarction (STEMI). We present two cases in which computed tomography angiography (CTA) was essential in identifying SOV dissection. In the first case, CTA confirmed a localized dissection of the right SOV. Surgical revascularization was delayed owing to initial diagnostic challenges and the family's hesitation, which ultimately led to a fatal outcome. In the second, intravascular ultrasound (IVUS) confirmed extrinsic compression of the right coronary artery (RCA), and following emergency stent implantation, coronary blood flow was restored, conservative treatment achieved a favorable clinical outcome. These cases highlight the pivotal role of early CTA when angiographic findings are incongruent with the clinical presentation, the utility of IVUS in determining the etiology of coronary artery occlusion, and the critical importance of timely revascularization.

## Introduction

Clinically, although STEMI secondary to aortic dissection (AD) is frequently encountered (1–3), STEMI caused by minor, localized SOV dissection represents an exceptionally rare and diagnostically challenging entity (4, 5). Patients may lack classic symptoms and signs of AD, but marked ST-segment elevation on ECG will still prompt immediate catheterization according to STEMI management protocols. Coronary angiography (CAG) and aortography may fail to detect minor SOV dissections (5). CTA provided a crucial diagnostic advantage by revealing aortic root pathology (6). Echocardiography and IVUS provided critical diagnostic clues suggestive of SOV dissection. Here, we report two cases of inferior STEMI caused by localized right SOV dissection, which had similar presentations but differing outcomes. CTA played a central diagnostic role in both cases and IVUS plays an important role in identifying atypical causes of ACS.

## Case presentation 1

**Figure 1:**
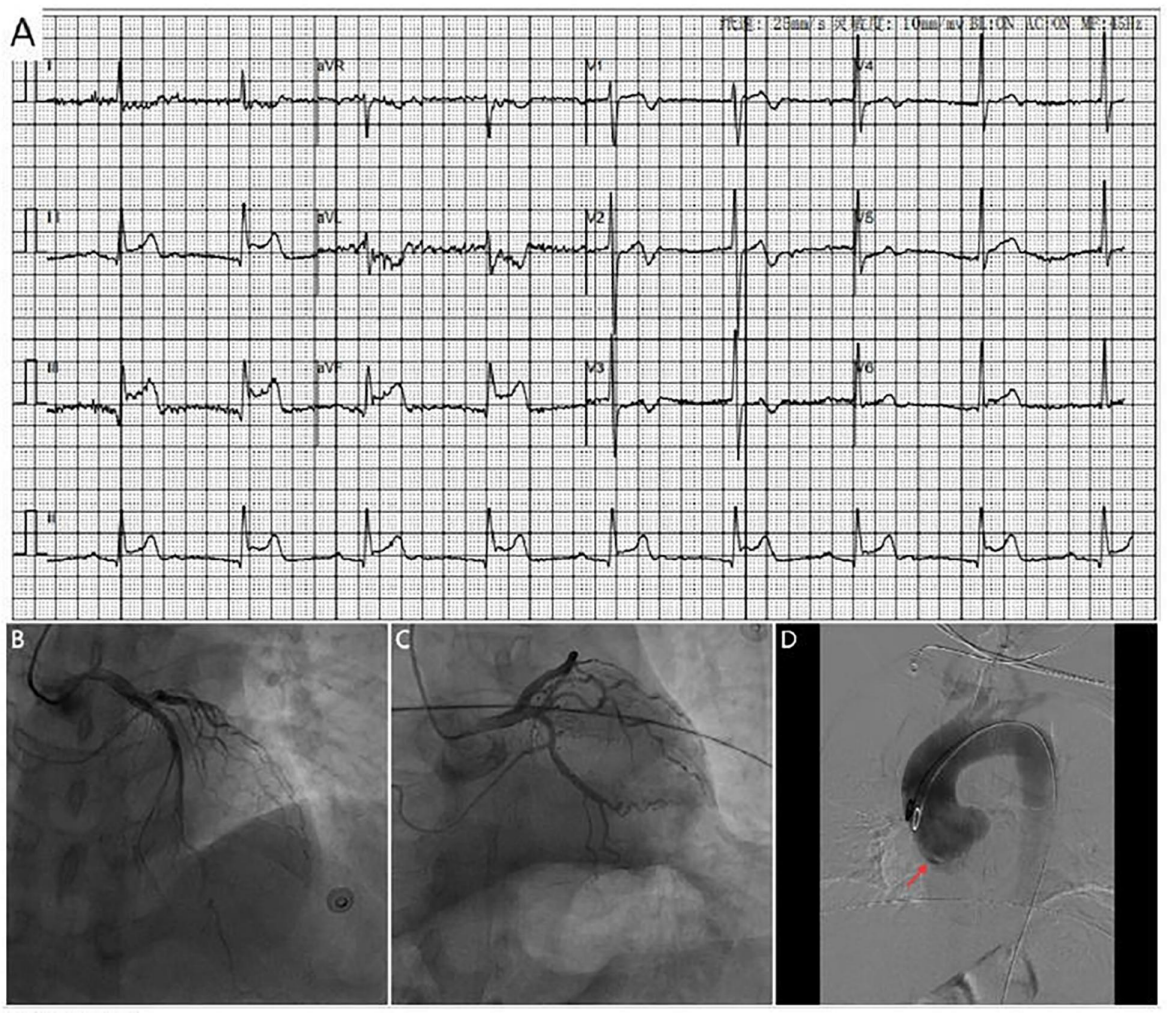
**(A)** ECG shows marked ST-segment elevation in leads II, III and avF, along with complete heart block **(B,C)**. Coronary angiography shows no significant stenosis in the left coronary artery. **(D)** Aortic angiography reveals possible dissection of the right coronary sinus of valsava (red arrow).

**Figure 2:**
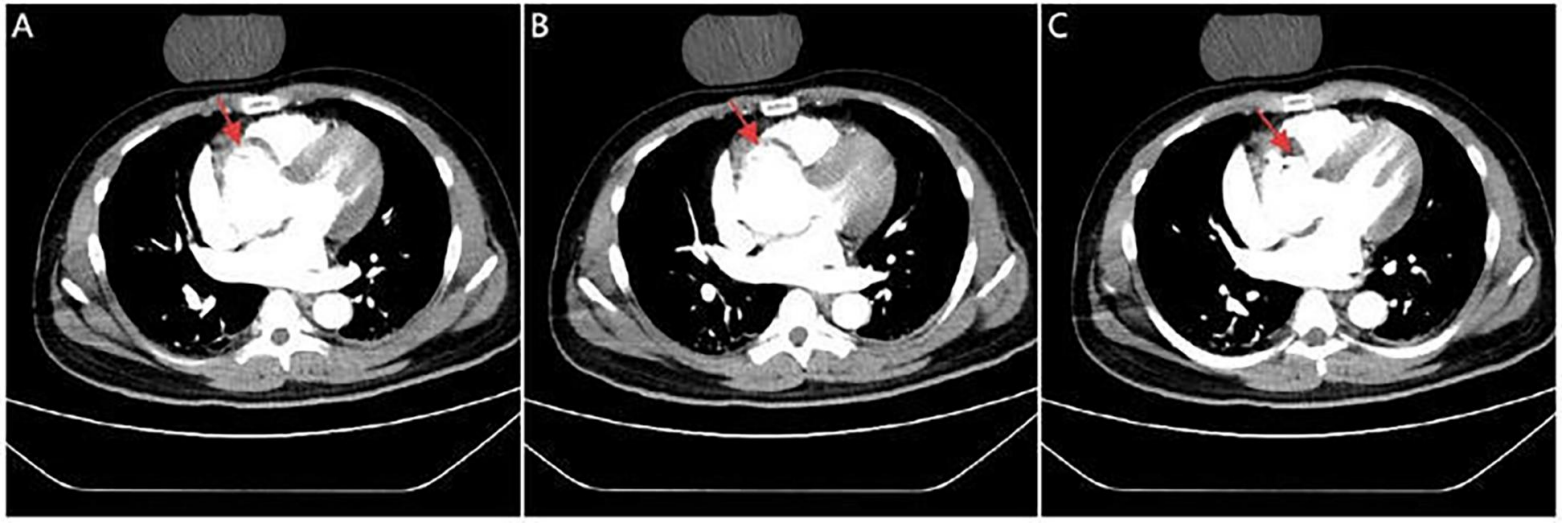
CTA images. Serial CTA images **(A–C)** show located right coronary sinus of valsava dissection (red arrow).

A 42-year-old man with no known comorbidities and a family history of AD presented with sudden-onset chest pain during esports gameplay. ECG showed inferior STEMI with complete heart block (Figure 1A). CAG revealed a normal left coronary system, but the RCA could not be engaged. Aortic root angiography showed no anomalous origin of the RCA; however, it suggested a possible dissection of the right SOV (Figures 1B–D; Supplementary Video 1). CTA revealed ascending aortic dilation and a localized dissection of the right SOV (Figures 2A–C; Supplementary Video 2). The emergency transthoracic echocardiography (TTE) revealed dilation of the ascending aorta, measuring 43 mm in diameter. Despite the recommendation for immediate surgery, family hesitation delayed intervention. The operative findings showed that a dissection of SOV extending into the RCA ostium. Coronary artery bypass grafting (CABG) and sinus reconstruction were performed, however the patient developed cardiogenic shock and refractory ventricular arrhythmias. The family declined mechanical circulatory support (MCS), and the patient subsequently died.

Timeline is shown in Supplementary Table 1.

## Case presentation 2

**Figure 3:**
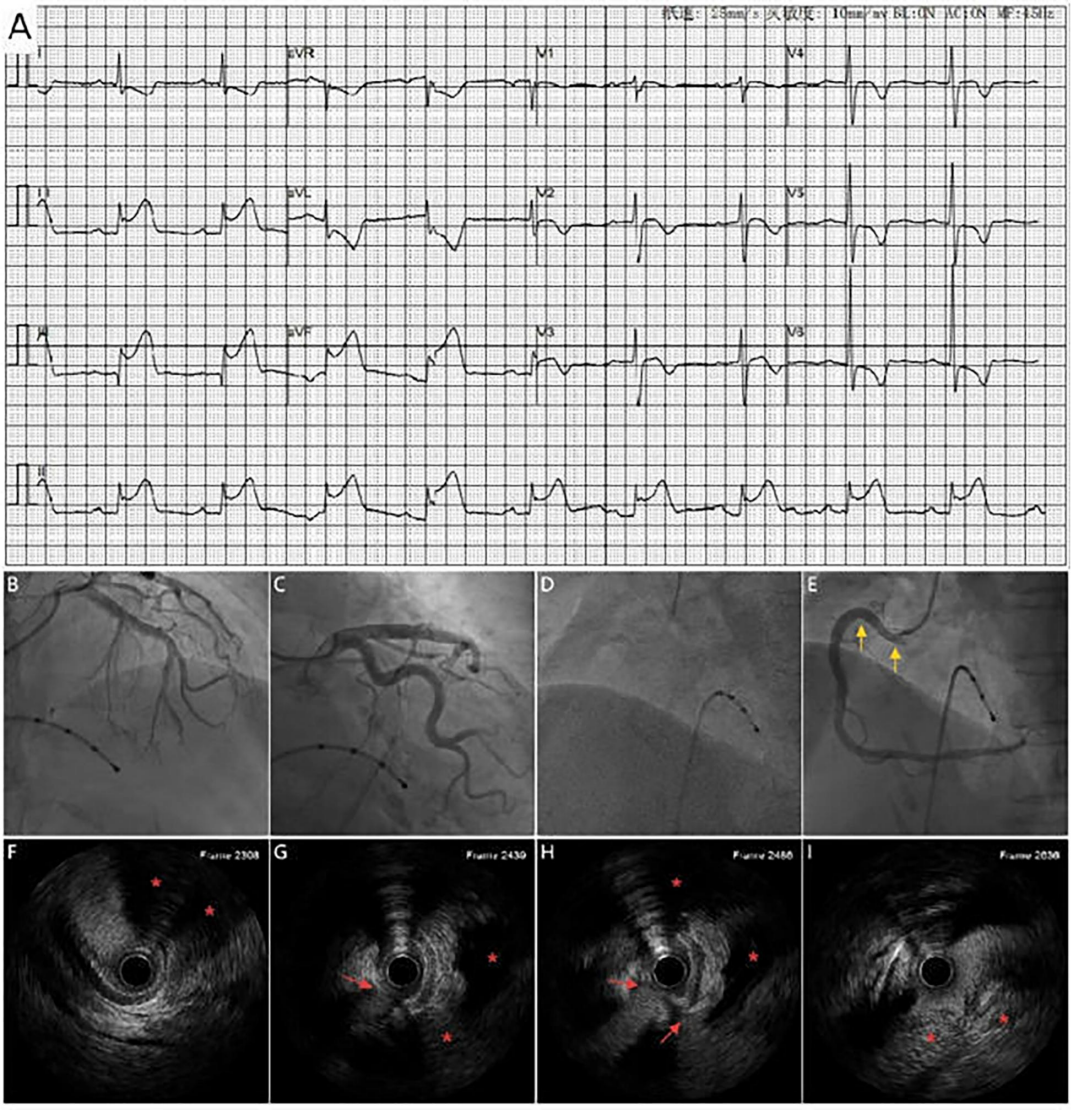
**(A)** ECG shows marked ST_segement elevation in leads II, III and avF, along with sinus bradycardia. Coronary angiography shows no significant stenosis in the left coronary artery **(B,C)** and complete proximal RCA occlusion **(D)**, A DES (yellow arrow) placement at the RCA ostium with restored antegrade flow **(E)**. **(F–I)** IVUS shows extravascular dissection (red arrow) and hematoma compressing the RCA (area marked with red asterisks).

**Figure 4:**
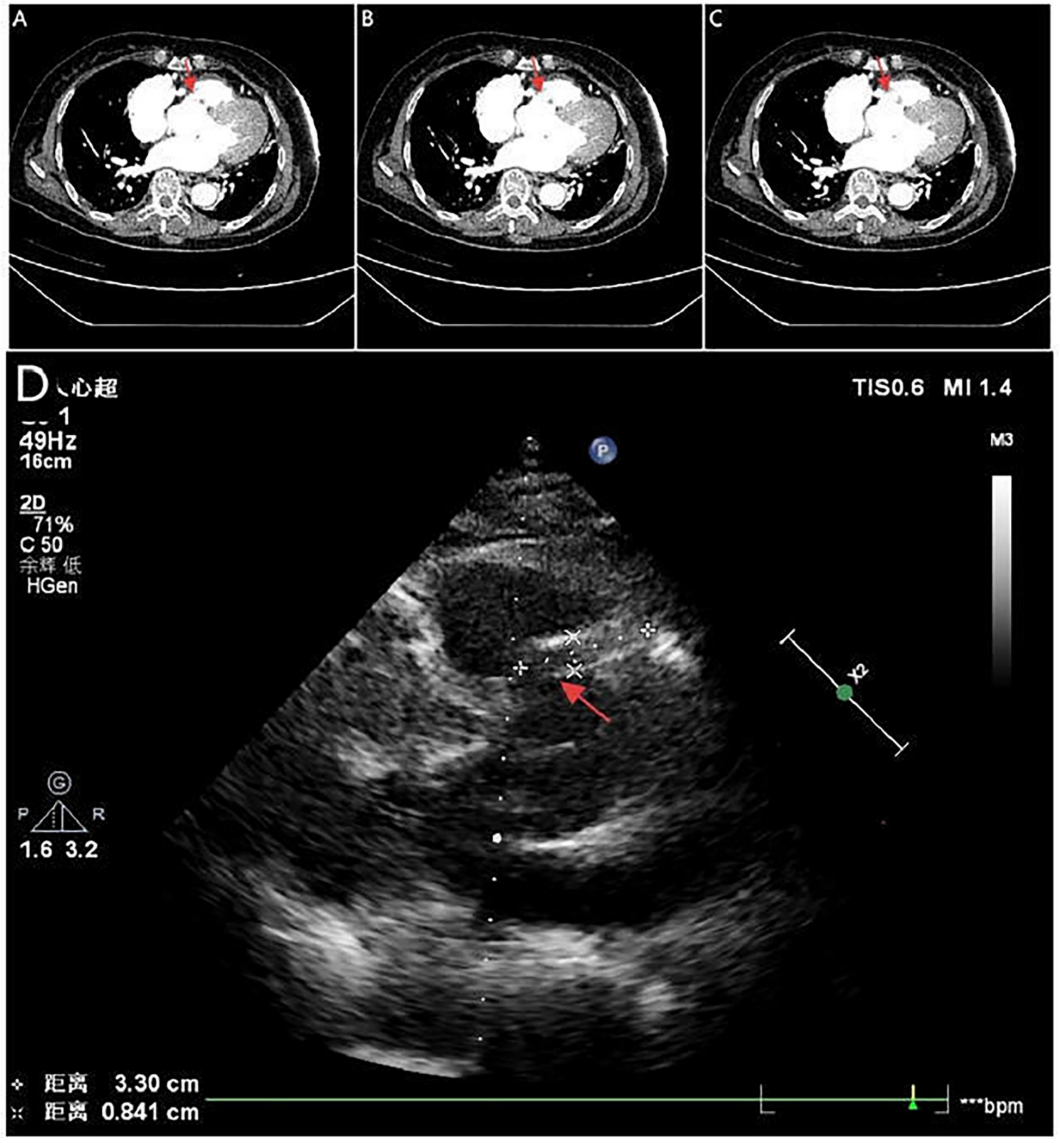
Serial CTA images **(A–C)** show located right coronary sinus of valsava dissection (red arrow). Echocardiography **(D)** demonstrates an intramural hematoma originating at the ostium of the RCA within the right coronary sinus, with partial extension into the proximal RCA (red arrow).

A 71-year-old woman with hypertension presented with chest tightness and diaphoresis. ECG revealed inferior STEMI and sinus bradycardia (Figure 3A). CAG demonstrated complete proximal RCA occlusion (Figures 3B–D; Supplementary Video 3) without visible intraluminal plaque on IVUS, but persistent extrinsic compression (Figures 3F–I; Supplementary Video 4). A drug-eluting stent (DES) was deployed at the ostium of the RCA with restored flow (Figure 3E). IVUS confirmed complete ostial coverage of the RCA and good stent apposition (Supplementary Video 6). CTA demonstrated mild aortic root dilation and right SOV dissection (Figures 4A–C; Supplementary Video 7). TTE demonstrated an intramural hematoma originating at the ostium of the RCA within the right coronary sinus, with partial extension into the proximal RCA (Figure 4D). The patient declined surgery and was managed conservatively. She remained stable with no recurrent symptoms during follow-up.

Timeline is shown in Supplementary Table 2.

## Discussion

It is uncommon for AD to initially present as STEMI, occurring in only 1%–2% of cases (7). Localized dissection of the SOV, a subtype of AD, likewise present with STEMI. This condition poses significant diagnostic challenges due to its nonspecific presentation and potential mimicry of STEMI (4). Etiologically SOV dissection can be categorized as either iatrogenic or spontaneous. Common iatrogenic causes include cardiac interventions (8) and intra-aortic balloon pump (IABP) catheterization (9). Spontaneous dissections are often associated with underlying conditions such as hypertension, congenital or genetic disorders (e.g., Marfan syndrome, Loeys-Dietz syndrome, bicuspid aortic valve) (10), vasculitides (e.g., Takayasu's arteritis) (11), and aortic atherosclerosis. Our cases showed no evidence of congenital or genetic disorders, nor of vasculitides.

Patients presenting with ST-segment elevation on ECG are typically managed according to STEMI protocols (3); however, dissection of SOV and STEMI require vastly different treatment approaches (12, 13). Diagnostic delay may occur when ischemic ECG changes mask aortic pathology (14, 15). Early recognition is critical, as inappropriate catheter-based intervention may delay definitive management and worsen outcomes (16). Thus, a high index of suspicion is essential when angiographic and clinical findings are discrepant.

A case of SOV dissection presenting as inferior STEMI was initially misdiagnosed as spontaneous coronary artery dissection (SCAD). Despite stenting, severe heart failure occurred. TTE revealed severe aortic regurgitation. Surgery confirmed dissection involving the RCA, and valve replacement led to recovery (5). Another two cases of spontaneous SOV dissection accompanied by severe aortic regurgitation were diagnosed by transesophageal echocardiography (TEE). CAG showed no evidence of coronary involvement in both cases. Surgical treatment resulted in good outcomes (17). Our cases showed typical STEMI on ECG but no abnormal signs of AD. CAG confirmed coronary involvement, highlighting the critical nature of these cases.

CTA, TTE/TEE, and IVUS all have important roles in the evaluation of SOV dissection (5, 17, 18). CTA offers rapid and detailed anatomical evaluation but may miss diagnoses in cases of suboptimal image quality. While TTE/TEE can identify dissection flaps and assess valvular function, comprehensive surgical planning still relies on CTA. In selected cases, IVUS may provide critical diagnostic clues—such as a dissection flap or extraluminal hematoma—prompting further imaging evaluation (5). Ultimately, integrating multiple modalities ensure accurate diagnosis and guides appropriate intervention.

Analysis suggests that entry-point stenting can achieve favorable clinical outcomes in cases of iatrogenic SOV dissection (8). However, spontaneous SOV dissection may necessitate urgent surgical management, including sinus reconstruction, aortic valve repair or replacement, and coronary revascularization (12, 19). Localized SOV dissection presenting as STEMI with concomitant coronary occlusion is exceedingly rare. The immediate priority is restoration of coronary flow. However, even when stenting achieves reperfusion, surgical repair may still be necessary if the intimal flap re-occludes the coronary ostium or results in severe aortic regurgitation (5).

In our first case, the patient was obese, had a family history of AD, and experienced emotional stress prior to symptom onset. Aortic intimal flap obstruction of the RCA ostium led to failed catheter engagement and precluded PCI (20). Emergent CTA confirmed right SOV dissection, representing an absolute indication for emergency surgery. Unfortunately, the patient died after CABG and sinus reconstruction due to recurrent malignant arrhythmias and refractory cardiogenic shock. The poor outcome was multifactorial, involving a nondominant left circumflex artery with right-dominant circulation, prolonged vessel occlusion causing extensive myocardial necrosis, delayed diagnosis, family hesitation delaying intervention, and refusal of postoperative MCS (21).

In the second case, the patient had long-standing hypertension and a normal coronary angiogram six years prior. IVUS clearly demonstrated extrinsic RCA compression by a hematoma extending into the perivascular space. Despite balloon angioplasty, no sustained TIMI 3 flow was achieved. In this setting, urgent coronary reperfusion was a critical step to stabilize the patient and create a window for potential aortic surgery (22, 23). This patient recovered without surgery. Serial CTA and echocardiography showed progressive regression of the right SOV dissection with septal echogenic band formation, indicating spontaneous healing. The favorable outcome of conservative management was attributable to a limited dissection entry and confined hematoma, the dual role of the stent in restoring perfusion and sealing the entry, strict hemodynamic control combined with close follow-up imaging surveillance (Supplementary Video 8). This case underscores the potential of conservative management in selected patients with restored coronary perfusion and anatomically stable lesions.

Both cases illustrate the diagnostic challenges of localized SOV dissection, particularly when RCA involvement obscures the true etiology. The SOV dissection may obstruct the coronary ostium with an intimal flap or cause extrinsic compression by a hematoma—features that CAG may not detect. In contrast, CTA was definitive in both cases, echocardiography contributed supportive evidence, and IVUS provided crucial mechanistic insight.

## Conclusion

Taken together, these cases emphasize the importance of maintaining diagnostic vigilance for SOV dissection in patients with STEMI-like presentations but atypical angiographic findings. Early use of imaging modalities such CTA, echocardiography and IVUS enables timely and accurate diagnosis. Moreover, individualized management strategies—including timely surgery when PCI fails, or conservative treatment when reperfusion is achieved—may optimize outcomes in this rare but life-threatening condition. Importantly, Close imaging follow-up is essential throughout the conservative management of SOV dissection.

## Data Availability

The original contributions presented in the study are included in the article/Supplementary Material, further inquiries can be directed to the corresponding author.
