# Supplementary material for: When the culprit lies outside the coronary artery: dual case report of coronary sinus of valsalva dissection presenting as STEMI
Source: Front Cardiovasc Med. 2025 Sep 22;12:1670164. doi: 10.3389/fcvm.2025.1670164 (PMC12497843; doi:10.3389/fcvm.2025.1670164)
Supplement: Supplementary file 1 [file Table1.pdf]

SUPPLEMENTARY TABLE 1 Timeline of clinical events

	Time	Time Point	Events
Day 1	15:40	Symptom Onset Time	
	16:12	Arrival at Hospital Gate	
	16:15	First Medical Contact	
	16:17	First ECG	ECG showed inferior STEMI
	16:44	Cardiac Catheterization Consent Obtained	
	16:55	Arrival in Cath Lab	
	16:58<br>18:40	Cardiac catheterization	Failed to cannulate the RCA
	18:59	CTA completed	CTA demonstrated right SOV dissection
	21:16	Surgical Consent Obtained	
	21:55	Transfer to Operating Room	
	23:20	Skin Incision	
	23:37	Sternotomy	
Day 2	00:05	CPB Initiation	
	00:25	Aortic Cross-Clamp	
	02:50	Aortic Cross-Clamp Off	
	03:40	CPB Termination	Hypotension
	04:20	Onset of Ventricular Fibrillation	Defibrillation, internal cardiac massage, and pharmacologic support
	04:50	Patient Deceased	
