# Supplementary material for: When the culprit lies outside the coronary artery: dual case report of coronary sinus of valsalva dissection presenting as STEMI
Source: Front Cardiovasc Med. 2025 Sep 22;12:1670164. doi: 10.3389/fcvm.2025.1670164 (PMC12497843; doi:10.3389/fcvm.2025.1670164)
Supplement: Supplementary file 2 [file Table2.pdf]

SUPPLEMENTARY TABLE 2 Timeline of clinical events

	Time	Time Point	Events
Day 1	07:20	Symptom Onset Time	
	08:20	Arrival at Hospital Gate	
	08:22	First Medical Contact	
	08:25	First ECG	ECG showed inferior STEMI
	08:48	Cardiac Catheterization Consent Obtained	
	09:20	Arrival in Cath Lab	
	09:31	Temporary Pacemaker Implanted	
	09:37	Cardiac Catheterization	RCA total occlusion
	09:43	Wire Crossing Time	
	10:18	Cardiac Catheterization Completed	Stent Implanted in RCA
	10:50	CTA completed	CTA demonstrated right SOV dissection
Day28	12:00	Discharged from hospital	
